# Development and validation of School Resilience Questionnaire (SRQ) in Iranian adolescents

**DOI:** 10.1186/s40359-023-01496-6

**Published:** 2024-01-18

**Authors:** Leila Afzali, Simin Hosseinian

**Affiliations:** Department of Counseling, Faculty of Education and Psychology, Alzahra University, Tehran, Iran

**Keywords:** Adolescent, Development, Resilience, School, Validation

## Abstract

**Background:**

School resilience, encompassing factors like teacher involvement and school supportiveness, is crucial for positive educational outcomes in adolescents. However, few validated scales measure school resilience. This study aimed to develop and validate the School Resilience Questionnaire (SRQ) for Iranian adolescents.

**Methods:**

The study used a cross-sectional design. The SRQ was developed through expert reviews, interviews with students, and evaluation of existing resilience measures. After expert feedback, the final 55-item questionnaire was used. Participants were high school adolescents from Tehran, Iran (2021–2022). A multi-stage cluster random sampling approach was used. Exploratory Factor Analysis (EFA) involved 200 students, and Confirmatory Factor Analysis (CFA) included 310 students to validate the factor structure. Convergent validity was assessed by examining correlations with academic support, while divergent validity was evaluated using academic burnout measures. Construct validity and reliability were also assessed.

**Results:**

EFA revealed six consistent factors across the sample. CFA confirmed significant and acceptable factor loadings for all SRQ items. Fit indices were RMSEA = 0.076; SRMR = 0.070, CFI = 0.94, NFI = 0.93, IFI = 0.94, RFI = 0.93. Convergent validity showed positive correlations between SRQ subscales and academic support. Divergent validity showed negative correlations between SRQ subscales and academic burnout (*p* < 0.05).

**Conclusion:**

The study successfully developed and validated the 55-item SRQ for Iranian adolescents. The questionnaire demonstrates satisfactory psychometric properties, making it a valuable tool for research and evaluation purposes in this context.

**Supplementary Information:**

The online version contains supplementary material available at 10.1186/s40359-023-01496-6.

## Introduction

Adolescence is a phase characterized by rapid physical, cognitive, and socioemotional growth, presenting a spectrum of challenges and opportunities [1]. Adolescents encountering such difficulties are at a heightened risk of facing adverse educational consequences, including peer rejection, academic underperformance, and potentially discontinuing formal education. Accordingly, acknowledging and addressing these challenges during this pivotal phase is crucial for enhancing the well-being and future prospects of adolescents [2]. Adolescence period often witnesses a decrease in parental involvement in adolescents’ education [3]. Such circumstances may increase adolescents' susceptibility to conduct-related challenges. Considering these factors, it is plausible to expect a correlation between the school environment and adolescents' socioemotional development. Consequently, it becomes essential to focus on adolescents' perceptions of the school climate and their sense of connection with the educational institution. This focus is crucial to attain a deeper understanding and provide effective support for their psychosocial growth [4].

Establishing a nurturing and supportive environment within educational institutions is crucial for the holistic development and well-being of adolescents. Schools play a vital role in this process by creating an atmosphere that values and supports students, thereby contributing to their academic, social, and emotional growth. The provision of guidance, encouragement, and adequate resources is pivotal in empowering students to navigate various challenges effectively [5]. Emphasizing the importance of the educational environment in fostering a positive school climate is essential. By prioritizing a nurturing atmosphere, educational institutions significantly enhance student engagement, motivation, and satisfaction with their educational experience, thereby strengthening their connection to the school [6].

Recognizing the substantial impact of the developmental environment, including the school setting, on the trajectory of children and adolescents is paramount [7]. The developmental environment has the potential to either exacerbate the negative effects of risk factors or provide protection to adolescents, ultimately shaping their developmental outcomes [8]. Schools, functioning as nurturing hubs, play a vital role in facilitating growth and progress by providing care and support to all learners and communities. Extensive research on resilience in schools consistently highlights the pivotal role that educational institutions play in the development of adolescents [9]. These studies consistently demonstrate that a significant number of adolescents can overcome the adverse impacts of environmental risks and emerge as successful, competent, and resilient individuals.

School resilience, a key concept in the educational community, comprises multiple dimensions that contribute to the resilience and mental well-being of adolescents. Research has highlighted the impact of various emotional, cognitive, behavioral, and social factors on youth mental health [10]. The school environment significantly influences these factors. Resilience is understood through multi-systemic [11, 12] and socio-ecological [13] frameworks, focusing on the interaction of individuals and systems, including relationships, meanings, and values. School resilience is thus seen as the development of these interconnected aspects within the educational setting, with the quality and nature of relationships within the school community playing a crucial role.

The development of a scale for assessing "school resilience" among adolescents is highly significant in academic and research contexts [14]. Such a scale provides a standardized and reliable means for measuring resilience in educational environments, enabling objective comparisons across various schools and student populations. This facilitates the identification of trends and contributes to a deeper understanding of resilience factors in educational settings. Additionally, the scale is instrumental in evaluating the effectiveness of resilience-promoting interventions and programs. A validated school resilience scale not only strengthens research credibility but also underpins academic success, career achievement, and psychological well-being in educational contexts [15, 16].

The cultivation of school resilience is vital for all educational stakeholders. It ensures equitable learning opportunities and positive outcomes for all students. Measuring school resilience is beneficial for guiding interventions involving various contributors, including teachers, students, parents, governmental bodies, businesses, and community leaders [14]. These efforts, which range from creating supportive environments to implementing resilience-building policies, have been shown to improve academic performance and reduce dropout rates [17]. These studies highlight highlights how a collaborative effort among teachers, parents, and community leaders resulted in enhanced student well-being and resilience in a low-income neighborhood.

Cultivating school resilience is vital for academic, career, and psychological well-being in education. Developing accurate resilience scales and enhancing school resilience are key to improving student outcomes and promoting evidence-based practices in educational resilience. The gap in reliable tools for measuring School Resilience led to our study's goal of creating a valid School Resilience Questionnaire (SRQ) for Iranian adolescents. This study entails developing and testing the SRQ's validity and reliability, starting with adapting 60 items from existing research, carefully selected and refined to ensure accuracy in assessing school resilience.

Recognizing the range of resilience assessment tools used by Iranian researchers [18], it's notable that these primarily address general aspects of adolescent resilience and may not fully encompass the unique facets of school resilience, particularly within the distinct cultural and educational context of Iranian adolescents. This highlights the need for a specialized instrument, the SRQ, specifically crafted to capture the complex nature of resilience in school environments. The SRQ is designed to enhance existing tools by offering a more thorough, culturally sensitive assessment, thereby deepening the understanding of the influence of school environments on the resilience and development of Iranian adolescents. Consequently, the SRQ's development addresses a significant gap in current research and provides a nuanced view of the educational environment's role in fostering resilience among Iranian youth.

It is also essential to acknowledge the presence of various resilience measurement scales utilized in Iran to assess adolescent resilience. These scales have been instrumental in understanding resilience among Iranian youth across diverse contexts. However, the unique educational, cultural, and situational challenges faced by Iranian adolescents necessitate the SRQ's development. Although existing scales offer valuable insights, the SRQ aims to address specific needs and experiences of Iranian adolescents within their educational settings. By building on the strengths of existing resilience measures and tailoring the SRQ to the particular needs and experiences of Iranian adolescents, this study ensures that the SRQ aligns with cultural and contextual factors influencing Iranian youth's resilience, thereby enhancing its relevance and effectiveness in this context.

This adaptation process drew upon the established developmental steps employed in School Resilience, aiming to ensure the high standards of measurement for school resilience in our adapted questionnaire. To optimize the questionnaire's clarity, coherence, and response consistency, we implemented modifications, including the utilization of 5-point Likert response format. This approach facilitates more nuanced and precise assessment of individuals' perspectives and attitudes regarding school resilience, effectively capturing the intended information while minimizing potential ambiguity in responses.

The current research on resilience measurement for adolescents reveals the absence of a culturally specific tool for assessing school resilience in Iranian adolescents. The SRQ addresses this gap, uniquely tailored to the Iranian context. Differing from existing scales, the SRQ is meticulously designed to align with the cultural and educational nuances of Iranian schools, ensuring accuracy and relevance. Its validity and reliability have been rigorously confirmed through various evaluations, solidifying its role as dependable tool in educational psychology. The SRQ is vital for understanding and enhancing Iranian adolescents' resilience, contributing to their well-being and academic success. This study's development and validation of the SRQ respond to the specific needs of Iranian youth, aiming to optimize resilience assessments and support students' overall development.

Previous research has extensively explored adolescent resilience and coping mechanisms in varied contexts, yet the specific aspect of school resilience in the Iranian adolescent context remains underexplored. The SRQ has been developed as a culturally relevant and specific tool to address this gap. Its development, validation, and implementation offer a significant contribution to assessing and enhancing school resilience among Iranian adolescents, thereby positively impacting their well-being and academic success. The SRQ's introduction addresses the need for a culturally sensitive instrument tailored to Iranian adolescents' unique educational experiences. This addition to the field enriches the ongoing dialogue on school resilience, providing a comprehensive tool for understanding and improving resilience in Iranian educational settings.

Through this research and the establishment of a valid School Resilience Questionnaire, our study's objective is to provide educators, researchers, and policymakers with an effective and reliable tool to assess and comprehend the levels of resilience among Iranian adolescents within the educational setting. This comprehensive understanding of school resilience holds significant potential in informing the development of targeted interventions, policies, and practices aimed at promoting the holistic well-being and academic success of students. Ultimately, the development of a valid School Resilience Questionnaire equips stakeholders with a reliable tool to assess and enhance the resilience of Iranian adolescents, leading to improved well-being and academic success.

## Methods

The present study employed a cross-sectional research design to investigate its objectives. Specifically, a comprehensive survey methodology was utilized to develop and validate the School Resilience Questionnaire (SRQ). To ensure the robustness of the study design, several rigorous steps were undertaken, as follows:

Firstly, a thorough literature review was conducted to gather relevant information and generate a pool of items for the SRQ. These items underwent assessment by an Expert Panel, and a meticulous selection process was carried out based on the valuable feedback provided. The selected items formed the preliminary scale, which served as the basis for further evaluation.

Subsequently, a pilot sample was utilized to assess the suitability of the preliminary scale and refine its design. This process aimed to enhance the clarity and effectiveness of the scale. Once the necessary refinements were made, the scale was administered to a larger sample for further validation.

To evaluate the construct validity of the SRQ, Exploratory Factor Analysis (EFA) was employed to investigate the underlying structure of the scale. Confirmatory Factor Analysis (CFA) was then conducted to assess the consistency and appropriateness of the identified factor structure.

In order to determine the internal reliability of the scale, Cronbach's alpha coefficient was calculated. This statistical measure ensures the consistency of the measurement of the construct being assessed. Additionally, test–retest reliability was examined to assess the stability of the SRQ over time.

Moreover, the convergent validity of the SRQ was evaluated by examining correlations between the SRQ and academic support. This analysis aimed to establish whether the SRQ captures similar aspects as other established measures in the field. Similarly, divergent validity was assessed by examining correlations between the SRQ and academic burnout. These comparisons were made to ascertain the scale's ability to differentiate between related and unrelated constructs.

### Item generation and content validity assessment

The principal investigator and co-investigator performed a comprehensive examination of the existing literature on school resilience, focusing specifically on the scales and other measurement tools employed in this field. Drawing upon the valuable insights obtained from this extensive review, we developed a meticulous set of items for our study. These items were carefully crafted as closed-ended declarative statements, employing a straightforward language style to ensure optimal comprehension among individuals with basic reading abilities. Following the methodological framework outlined in the methods section, we developed 60 items.

To ensure the generation of representative and clear items, all the items underwent thorough discussions with the authors. Subsequently, all of items garnered unanimous agreement was chosen for inclusion. The qualitative findings and items underwent meticulous review by three experts well versed in the realms of educational psychology and education literature. Their expertise helped to assess the appropriateness of the questions. It is noteworthy that, based on the experts' feedback, 5 questions were deleted and 1 question was modified. After this, a panel comprising nine specialists in educational psychology undertook the evaluation of the items to ascertain their indispensability in assessing the specific domain of interest. Employing Lawshe’s [19] formula, the panel calculated the content validity ratio (CVR) for each item, gauging its content validity. This rigorous evaluation process, characterized by extensive collaboration with authors and expert input, served to fortify the comprehensiveness and integrity of the research instrument:

$$CVR=\frac{ne-{\displaystyle\frac N2}}{\displaystyle\frac N2}$$

(ne= number of experts who considered items ‘essential’, N= total number of experts)

The Content Validity Ratio (CVR) should be equal to or above 0.78 for each item [19] and we had involved the participation of 9 panels of experts. In the present study, none of the items was eliminated in this phase, as all of them exhibited a CVR equal to or above 0.78. Moreover, to assess the relevance of the items, the Content Validity Index (CVI) was calculated. Unlike CVR, which is calculated individually for each item, CVI is computed for the entire scale and encompasses two types: item-level CVI (I-CVI) and scale-level CVI (S-CVI). The experts provided ratings for each item using a 4-point Likert scale, ranging from "1 = not relevant" to "4 = quite relevant". To calculate I-CVI, the total number of experts divided the number of experts who rated an item as 3 or 4. Some researchers recommended a minimum I-CVI of 0.78 with a panel of eight experts that we used 9 experts [20]. There are two approaches to calculate the S-CVI: S-CVI/UV (based on universal agreement) and S-CVI/Ave (based on the average method). When using the average method, there are two ways to calculate S-CVI: 1) by summing the I-CVI scores and dividing by the number of items, and 2) by summing the proportion of relevance ratings and dividing by the number of experts [21, 22]. In our questionnaire, all items demonstrated an I-CVI higher than 0.78. The calculation of S-CVI/Ave (based on I-CVI) resulted in a value of 0.94, indicating a satisfactory level of content validity. So after careful consideration and incorporation of the experts' feedback, we retained 55 items from the original pool of 60. Each item was associated with a response set ranging from 1 to 5, ("strongly disagree" (1), "disagree" (2), "neither agree nor disagree" (3), "agree" (4), and "strongly agree" (5)), resulting in a potential total score ranging from 55 to 270. This scoring system reflects the comprehensive nature of the measurement scale employed.

### Pilot test administration and feedback

The pilot examination of the emergent School Resilience Questionnaire (SRQ) was carried out on a convenience sample of participants. In addition to completing the survey, participants were invited to engage in telephone interviews to provide their valuable feedback. The pilot convenience sample predominantly consisted of adolescent students (*n* = 40) with mean age of 13.6 years.

Subsequent follow-up telephone interviews revealed unanimous consensus among the pilot participants regarding the clarity and comprehensibility of the questionnaire items. This participant feedback serves as clear evidence of the initial clarity and understandability of the SRQ. Finally, by utilizing these rigorous indices, we can state that the final items of scale were 55.

### Setting, participants, and data collection

Participants were recruited from Tehran, the capital of Iran, during the academic year 2021–2022, employing a multi-stage cluster random sampling approach, which is commonly used in social science research to ensure representative samples. This approach allows for the inclusion of diverse individuals from different districts, enhancing the generalizability of the findings within the specified academic field of study.

Under the guidelines proposed for scale development studies, it is recommended to employ a sample size ranging from 300 to 450 participants [23]. Aligning our method with these established recommendations, we conducted a distribution of 510 questionnaires among the targeted sample population in Tehran City, Iran. This approach ensures that our study adheres to rigorous standards and facilitates the attainment of statistically significant and reliable results within the context of our research.

Tehran City was divided into five distinct districts: North, South, Central, West, and East. From each district, one representative district and two educational units comprising first and second-secondary schools were selected. This methodological choice was made to capture a comprehensive view of the educational landscape in Tehran and to ensure a diverse representation of students from various regions of the city.

Questionnaires were then distributed among the students in the selected schools, following rigorous procedures to minimize bias and ensure the confidentiality of the responses. The randomly chosen regions included 2 (from the north),3 (from the center), 3 (from the west), 3 (from the east), and 3 (from the south). This distribution aimed to provide a balanced representation of the different districts and increase the diversity of the sample.

Strict inclusion criteria were applied to ensure the integrity of the participant selection process. Eligibility was limited to students studying in the first secondary school (7, 8, and 9) and second secondary school (10, 11, and 12), as these grades are crucial for capturing the targeted research outcomes within the specified academic discipline. Additionally, participants with physical or mental illnesses were excluded to ensure the validity and reliability of the collected data. The research team remained vigilant in monitoring the emotional well-being of the participants and provided necessary support or resources in cases of distress or discomfort.

In total, 510 individuals, comprising 249 boys (48.8%) and 261 girls (51.2%), were recruited for the study. The mean age ± standard deviation (SD) was calculated as 15.48 ± 1.69. The selected sample size allowed for a robust analysis and precise estimation of the research findings within the specified academic field.

The EFA involved the completion of the survey by 200 high school adolescents. This analysis aimed to explore the underlying factors and relationships among the measured variables. The CFA encompassed the responses of 310 adolescents to the questionnaires and focused on validating the proposed factor structure identified through the EFA. By employing both EFA and CFA, the study ensured a comprehensive examination of the research constructs and increased the reliability and validity of the findings within the specified academic discipline.

### Measures

- *The School Resilience Questionnaire (SRQ)****.*** The response set for each item in the study was determined through an expert panel Review process, conducted by a panel of esteemed experts in the field. Their expertise in educational psychology and education were instrumental in assessing the appropriateness of the questions. They carefully evaluated and reviewed the items to ensure their relevance and appropriateness for the research objectives. To enhance the validity and reliability of the response set, the panel considered various factors, including the wording of the items and the scale on which participants would respond. Ultimately, a 5-point Likert scale was selected, consisting of the following response options: “Strongly False”(1), “False(2)”,”Neither True nor False” (3),”True”(4), Strongly True(5). This scale was chosen to provide participants with a structured and standardized means of expressing their level of agreement or disagreement with each item. The decision to adopt a 5-point Likert scale was based on its wide usage in previous studies within the same academic discipline. This scale allows for nuanced responses while maintaining simplicity and ease of interpretation. Moreover, it aligns with established conventions and best practices in the field.
- *Academic Support Scale (ASS)*. This developed by Sands and Plunkett in 2005, was employed as an established instrument for assessing perceived academic support from various significant others, including mothers, fathers, teachers, and teenage friends. The selection of this scale was based on its well-documented efficacy in capturing the multifaceted nature of support within academic contexts. The scale consisted of 24 carefully crafted items, strategically divided into four subscales, with each subscale comprising six items. These subscales corresponded to the distinct sources of support: mothers, fathers, teachers, and teenage friends. Participants were provided with a four-point Likert scale, ranging from 1 (strongly disagree) to 4 (strongly agree), to indicate their agreement level with each item. To ensure a comprehensive assessment, mean scores were computed for each subscale by aggregating responses to the respective six items. This approach allowed for a nuanced evaluation of support received from different significant others. The reliability of the ASS was established through a pilot study involving a sizeable sample of participants, although the precise number is not mentioned. The reported Cronbach's alpha coefficients ranging from 0.89 to 0.93 indicate the internal consistency and stability of the scale. [24]. In the current study, the ASS was administered using a standardized administration procedure, and Cronbach's alpha coefficients for the subscales measuring support from mothers, fathers, teachers, and teenage friends were found to be 0.90, 0.98, 0.78, and 0.83, respectively.
- *The Academic Burnout Scale* [25]. This was developed in 2007 to comprehensively assess the levels of burnout among students within an academic context. This scale, comprising 15 items, was designed to capture three distinct dimensions: emotional exhaustion (consisting of 5 items), cynicism (comprising 4 items), and academic efficacy (including 6 items). Participants were required to express their degree of agreement with each item using a Likert response scale ranging from 1 (totally disagree) to 4 (totally agree). The selection of the Academic Burnout Scale was motivated by the critical need to address and understand burnout among students. Its multidimensional approach enables a comprehensive assessment of the various facets of burnout experienced in educational settings. In the specific context of research conducted in Iran, reliability coefficients were calculated for the three dimensions of emotional exhaustion, cynicism, and academic efficacy. These coefficients were found to be 0.70, 0.82, and 0.75, respectively, indicating good internal consistency [26]. However, further details regarding the sample size and participant characteristics were not provided, limiting the generalizability of the findings. The Academic Burnout Scale has been widely employed in academic research to investigate and quantify burnout among students. Furthermore, in the present study, the Cronbach's alpha coefficients for emotional exhaustion, cynicism, and academic efficacy were 0.72, 0.79, and 0.80, respectively, indicating satisfactory reliability.

### Ethical considerations

Ethical considerations played a fundamental role in the research process, with stringent measures implemented to protect the well-being and rights of the participants. The study received ethical approval from the ethics committee at Tehran University, which ensures that research proposals adhere to established ethical guidelines and promotes transparency and credibility. Written informed consent was carefully obtained from all participants/ families or legal guardians, who were provided with comprehensive information regarding the study's purpose, procedures, potential risks, and benefits. Participants were assured of their voluntary participation and given the freedom to withdraw from the study without facing any adverse consequences. Emphasizing the significance of informed consent demonstrates respect for autonomy and ethical conduct. Privacy and confidentiality were prioritized throughout the research process. Participants were guaranteed that their personal information would be treated confidentially and used solely for the purposes of the study. Stringent data anonymization techniques were employed to ensure that no identifiable information would be disclosed in any publications or reports resulting from the study. This safeguard helps maintain participants' anonymity and confidentiality. To minimize potential harm, additional measures were taken to address the emotional well-being of the participants. Debriefing procedures were implemented, and participants were provided access to support resources in case of any distress or discomfort arising from their involvement in the study. Ensuring the participants' welfare further strengthens the ethical foundation of the research. In summary, ethical considerations in this study encompassed obtaining informed consent, ensuring privacy and confidentiality, minimizing harm to participants, and adhering to the ethical guidelines established by the ethics committee at Tehran University. These rigorous measures were implemented to uphold ethical standards, protect participants' rights, and contribute to the integrity of the research process.

### Statistical analysis

Descriptive statistics were calculated, presenting categorical data in the form of counts and percentages. To assess the content validity of the questionnaire, we employed the Content Validity Index (CVI) and Content Validity Ratio (CVR), considering values exceeding 0.7 as indicative of a high level of content validity [27]. Once it was determined that the data met the assumptions for factor analysis, we divided the sample randomly into two groups: one for the EFA and the other for the CFA [28]. For the EFA, we utilized IBM SPSS Statistics 24.0 software (IBM SPSS Statistics, Inc., Armonk, USA). The results of the EFA informed the hypothesis of the CFA model, which we evaluated using Amos 26 software (IBM® SPSS® Amos TM26). We employed the Maximum Likelihood approach for parameter estimation. To assess the goodness of fit of the resulting factor structure, we used six fit indices: root mean square error of approximation (RMSEA; criterion ≤ 0.05), goodness of fit index (GFI; criterion > 0.08), relative fit index (RFI; criterion > 0.99), normed fit index (NFI; criterion > 0.99), and incremental fit index (IFI; criterion > 0.90) [29]. These indices were used to determine the adequacy of the factor model. The reliability of the measurements was evaluated using Cronbach's alpha index, a widely recognized measure of internal consistency. This index assesses the degree of correlation among the items within the measurement instrument, providing valuable insights into the data's reliability. By employing these rigorous statistical procedures, we ensured a comprehensive evaluation of the questionnaire's content validity, factor structure, goodness of fit, and reliability. These analytical methods contribute to the robustness and accuracy of our research findings.

## Results

### Exploratory Factor Analysis (EFA)

The internal structure of SRQ in Iranian students was tested by EFA using Principal Component Analysis (PCA) with varimax rotation to assess level of conformity and assign names to the extracted factors. The Kaiser- Meyer-Olkin (KMO) test was 0.90, above the recommended value of 0.6 [30]. The Kaiser–Meyer–Olkin index, a reliable measure for EFA, yielded a value of 90%, indicating good sampling adequacy. The skewness and kurtosis indexes were between 3 and -3, so it can be said that the data is normal. Subsequently, the remaining six factors underwent orthogonal rotation using the Varimax method to facilitate factor naming and estimation through the likelihood method. Bartlett's Test of Sphericity reached statistical significance (× 2 = 14,188.27, *p* < 0.001), indicating that the data were suitable for factor analysis. The initial analysis results showed six factors with eigenvalues greater than 1, which explained 73.06% of variance.

**Fig. 1:**
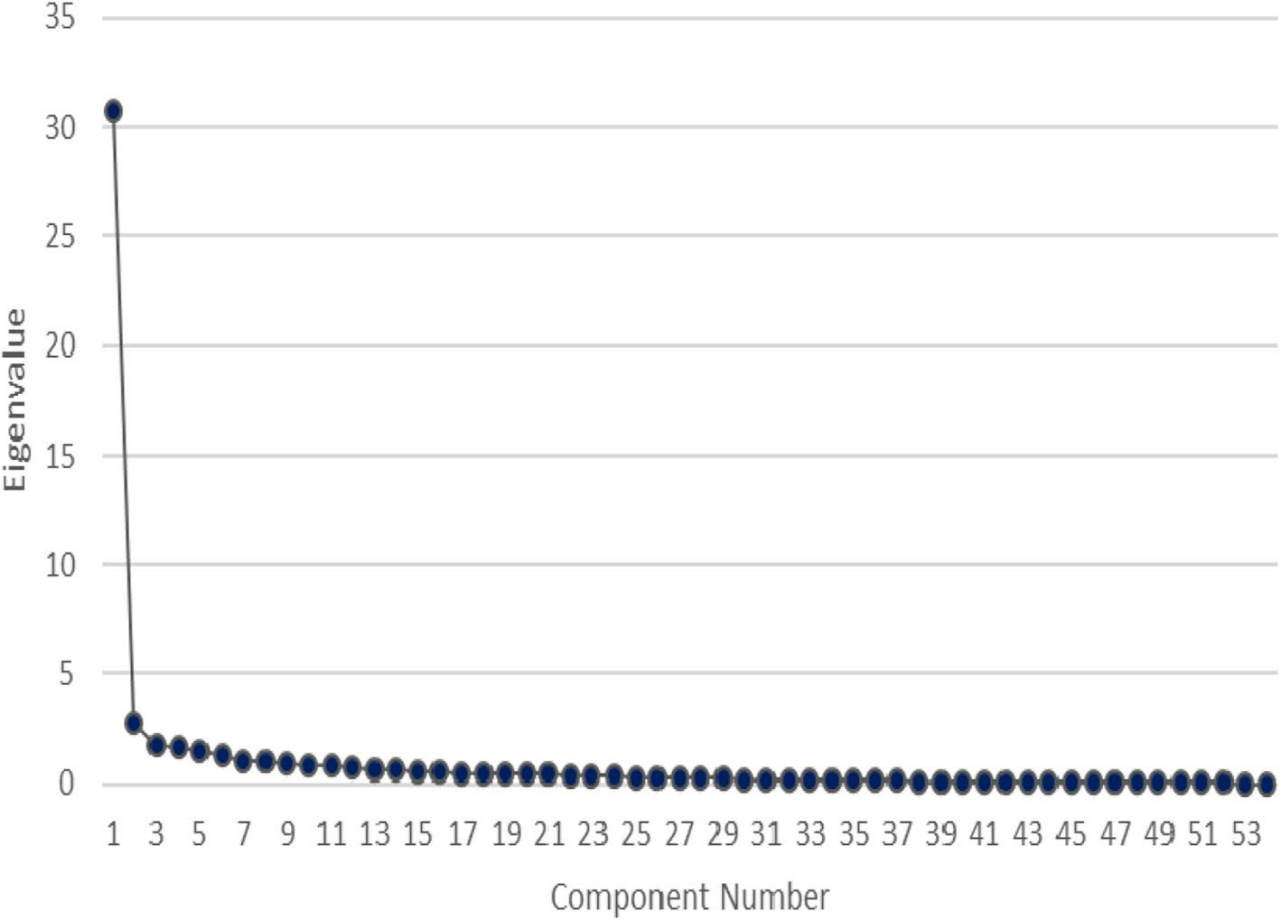
Scree plot

However, inspection of Scree Plot showed a clear break after the six components (Fig. 1). This was also supported by results of parallel analysis that showed six components with eigenvalues above the corresponding criterion values for randomly generated data matrix of same size (55 variables × 200 respondents). These six components explained 73.06% of the variance. PCA also showed that all factor loadings on six factors were above 0.50 (Table 1).

**Table 1:** EFA of 55-items of school Resilience Questionnaire (SRQ)

	M	SD	Factor Loading	Cronbach's Alpha if Item Deleted	Corrected Item-Total Correlation	**Eigenvalue**		
						Total	% of variance	Cumulative %
Q1	3.21	1.542	.719	.985	.723	30.723	56.895	56.895
Q2	4.11	1.158	.676	.985	.645	2.788	5.163	62.058
Q3	3.80	1.447	.799	.985	.806	1.787	3.309	65.367
Q4	3.71	1.465	.758	.985	.745	1.624	2.899	68.266
Q5	3.59	1.583	.848	.984	.814	1.508	2.608	70.874
Q6	3.67	1.518	.856	.984	.836	1.302	2.190	73.064
Q7	3.77	1.582	.847	.985	.804	.987	2.140	75.204
Q8	3.71	1.561	.834	.985	.783	.978	2.037	77.241
Q9	3.86	1.524	.735	.985	.741	.958	1.775	79.016
Q10	3.44	1.600	.818	.984	.830	.864	1.600	80.616
Q11	4.03	1.301	.718	.985	.742	.822	1.522	82.138
Q12	3.85	1.429	.715	.985	.748	.695	1.287	83.425
Q13	3.60	1.500	.715	.985	.689	.606	1.122	84.547
Q14	3.59	1.541	.805	.985	.786	.599	1.110	85.656
Q15	4.01	1.307	.804	.985	.809	.583	1.080	86.736
Q16	3.40	1.595	.830	.984	.823	.525	.973	87.709
Q17	3.64	1.477	.859	.984	.884	.480	.889	88.597
Q18	3.20	1.647	.768	.985	.693	.442	.819	89.416
Q19	3.41	1.570	.721	.985	.537	.428	.793	90.209
Q20	3.61	1.459	.766	.985	.811	.407	.754	90.963
Q21	3.76	1.488	.872	.985	.812	.400	.741	91.703
Q22	3.93	1.334	.758	.985	.793	.376	.696	92.399
Q23	3.42	1.522	.678	.985	.423	.314	.582	92.982
Q24	3.81	1.370	.781	.984	.836	.307	.568	93.550
Q25	3.71	1.398	.773	.984	.855	.297	.550	94.100
Q26	3.31	1.531	.685	.985	.704	.283	.524	94.624
Q27	3.40	1.480	.716	.985	.768	.252	.467	95.091
Q28	2.87	1.717	.801	.985	.736	.237	.439	95.530
Q29	3.42	1.654	.670	.985	.676	.228	.421	95.951
Q30	2.91	1.743	.794	.985	.666	.198	.367	96.318
Q31	3.82	1.314	.704	.985	.586	.194	.359	96.677
Q32	3.57	1.486	.714	.985	.441	.184	.341	97.018
Q33	3.80	1.330	.793	.985	.652	.166	.308	97.326
Q34	3.77	1.466	.720	.985	.623	.155	.286	97.612
Q35	3.61	1.469	.808	.985	.599	.142	.263	97.874
Q36	3.19	1.611	.797	.985	.766	.121	.224	98.099
Q37	3.24	1.608	.797	.985	.672	.119	.221	98.320
Q38	3.02	1.683	.709	.985	.701	.112	.208	98.528
Q39	3.40	1.626	.758	.985	.693	.097	.179	98.707
Q40	3.69	1.587	.793	.984	.821	.093	.172	98.879
Q41	3.68	1.439	.769	.985	.809	.083	.154	99.033
Q42	3.81	1.437	.754	.984	.823	.078	.145	99.179
Q43	3.96	1.389	.666	.985	.546	.069	.127	99.306
Q44	2.94	1.682	.699	.985	.645	.066	.122	99.428
Q45	3.04	1.659	.795	.985	.662	.052	.097	99.524
Q46	3.23	1.631	.834	.985	.761	.047	.087	99.611
Q47	3.55	1.562	.789	.985	.752	.040	.074	99.685
Q48	3.46	1.556	.815	.984	.859	.036	.066	99.751
Q49	3.28	1.659	.820	.984	.821	.031	.058	99.809
Q50	3.43	1.630	.804	.985	.781	.028	.051	99.860
Q51	3.78	1.495	.777	.985	.798	.024	.044	99.904
Q52	3.75	1.500	.864	.984	.841	.021	.040	99.944
Q53	3.71	1.513	.850	.985	.811	.017	.032	99.976
Q54	3.91	1.462	.752	.983	.784	.014	.028	99.981
Q55	3.91	1.413	.762	.985	.796	.013	.024	100.00

We conducted EFAs to assess the scale’s underlying structure using IBM SPSS Statistics 26.0 (IBM SPSS Statistics, Inc., Armonk, USA) software. Our EFA sample comprised 200 participants.

### Construct validity

In this study, the validity of the structure was evaluated using the CFA. The factor analysis was conducted using the entire sample size of 200 observations, resulting in the identification of six factors that accounted for over 73% of the variance. Based on the factor loadings and the content of the questions, the factors were named as follows: Factor 1—"Teacher's Skills Creating Resilient Classroom," Factor 2—"Collaborative Environment," Factor 3—"Departmental Security Environment," Factor 4—"Intimate Environment," Factor 5—"Respectful Environment," and Factor 6—"Legal Environment." Table 2 provides summary of the information along with the factor loadings.

**Table 2:** Factors extracted from EFA by Varimax rotation

Items	F1	F2	F3	F4	F5	F6
2	0.52					
3	0.68					
4	0.69					
5	0.82					
6	0.77					
7	0.75					
8	0.73					
10	0.66					
11	0.64					
13	0.50					
14	0.74					
15	0.72					
16	0.51					
17	0.66					
18	0.54					
20	0.56					
24	0.58					
40	0.55					
41	0.57					
42	0.67					
51	0.59					
52	0.75					
53	0.79					
54	0.69					
55	0.61					
26		0.43				
27		0.44				
31		0.67				
32		0.80				
33		0.87				
34		0.61				
35		0.81				
1			0.50			
9			0.52			
12			0.48			
21			0.47			
25			0.45			
30			0.46			
37			0.68			
38			0.47			
49			0.44			
50			0.55			
28				0.41		
29				0.47		
36				0.63		
45				0.65		
46				0.73		
47				0.49		
48				0.51		
19					0.47	
39					0.52	
43					0.75	
44					0.43	
22						0.50
23						0.76

*F1* Teacher's Skills Creating a Resilient Classroom, *F2* Collaborative Environment, *F3* Departmental Security Environment, *F4* Intimate Environment, *F5* Respectful Environment, *F6* Legal Environment

### Confirmatory Factor Analysis (CFA)

CFA was employed to assess the goodness of fit of the six-factor solution of the School Resilience Questionnaire (SRQ) using maximum likelihood estimation. A sample of 310 adolescents was utilized for the CFA analysis. The initial CFA was conducted with the six-factor solution and the final set of 55 items derived from the previous EFA.

The final CFA confirmed the presence of six factors and their relationship to 55 items. The factor loadings and fit statistics were obtained and are presented in Table 3. The explained variance, measured as partial R-squared, accounted for 68.25% of observed variance.

**Table 3:** CFA goodness fit index

Index	CFI	SRMR	NFI	IFI	RFI	RMSEA
Observed	0.94	0.070	0.93	0.94	0.93	0.076
Acceptable Range	0.9 >	< 0.08	0.9 >	0.9 >	0.9 >	< 0.08

*CFI* Comparative Fit Index, *SRMR* Standardized Root Mean Square Residual, *NFI* Normed Fit Index, *IFI* Incremental Fit Index, *RMSEA* Root Mean Square Error of Approximation

The CFA analysis aimed to assess the factorial structure of the questionnaire by comparing the hypothesized model to empirical data's covariance matrix. The goodness of fit results, shown in Table 3, indicated excellent fit for the data according to the findings of the CFA.

**Fig. 2:**
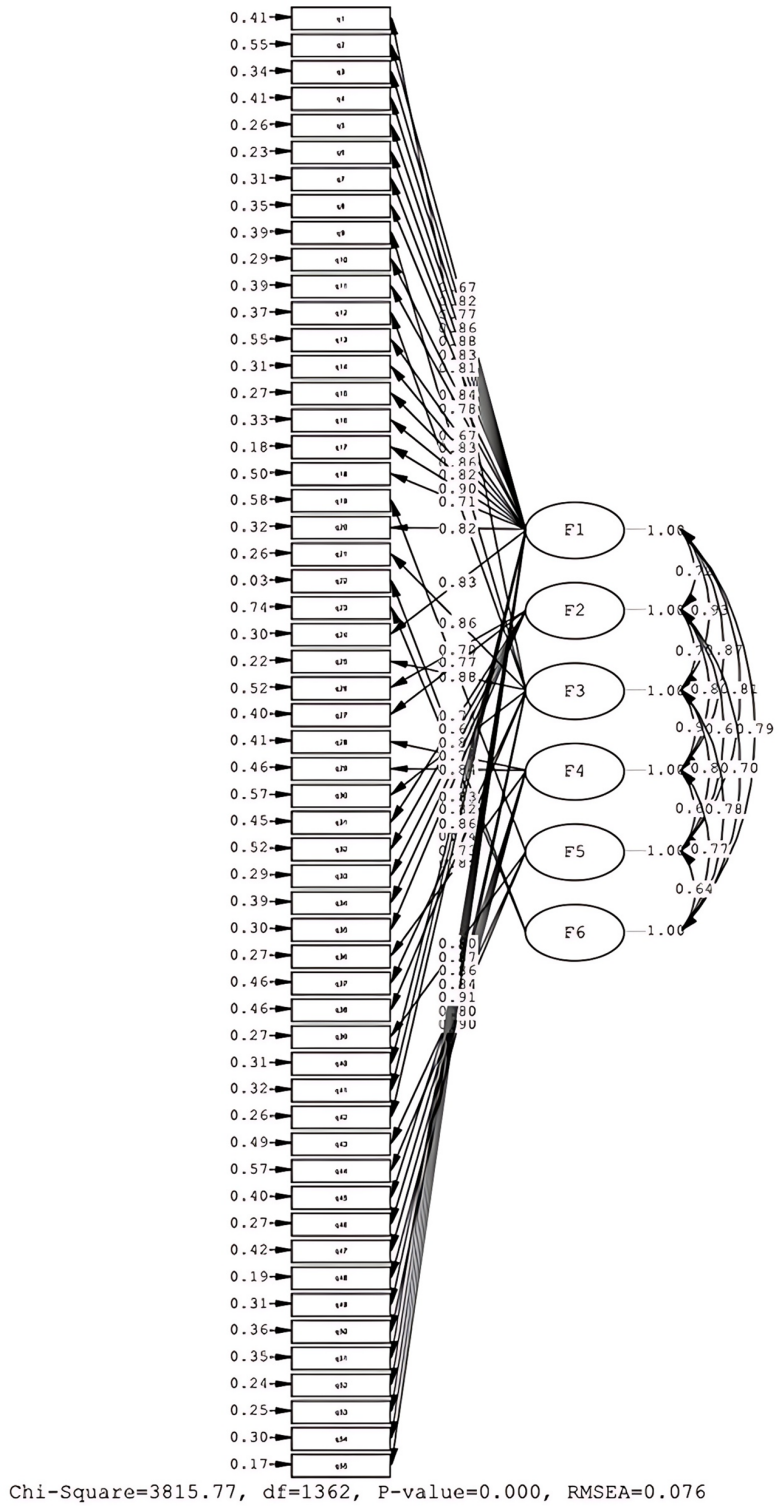
Confirmatory Factor Analysis for six-factors model of SRQ in Iranian Adolescents

The CFA results for a six-factor structure are shown in Table 3. These results are acceptable because the factor loadings of all items were significant and all items except item 6 were above 0.50. Model fit was estimated using the following fit indices: root mean square error of approximation (RMSEA; criterion 0.08) and its confidence interval 90%, Standardized Root Mean Square Residual (SRMR; criterion 0.08), Comparative Fit Index (CFI; criterion 0.90), Normed Fit Index (NFI; criterion 0.90), Incremental Fit Index (IFI; criterion 0.90), Relative Fit Index (RFI; criterion 0.90). The CFA results also showed that the six-factor structure provided a good fit to the data. In the present study, the fit indices of the model were RMSEA = 0.076; SRMR = 0.070, CFI = 0.94, NFI = 0.93, IFI = 0.94, RFI = 0.93. All items of the loadings showed a significant factor as shown in Table 3 and Fig. 2.

### Internal consistency reliability

The internal consistency reliability of the SRQ was determined using Cronbach's alpha for all participants and was 0.98, also McDonald's Omega was calculated 0.98 for total items. The internal consistency of the questioner using Cronbach's alpha for all components reported in Table 4, according to values higher than 0.7 for Cronbach's alpha values (except for the legal environment item), it is possible to ensure the appropriate reliability of this questionnaire.

**Table 4:** The Cronbach's alpha and Guttman Split-Half Coefficient of questioner

Subscale	Number of questions	Cronbach’s alpha	McDonald's Omega	Guttman Split-Half Coefficient
Teacher's Skills Creating a Resilient Classroom	24	0.97	0.97	0.96
Collaborative Environment	7	0.90	0.87	0.88
Departmental Security Environment	10	0.94	0.90	0.90
Intimate Environment	7	0.92	0.88	0.91
Respectful Environment	4	0.81	0.81	0.80
Legal Environment	2	0.76	0.60	0.61
Total	55	0.98	0.98	0.97

### Follow-Up study and test–retest reliability

Temporal stability was evaluated using a test–retest strategy in a small subsample of 125 participants from the main study. These participants were randomly selected and were asked to complete the SRQ again after a two-week interval. The results revealed a calculated test–retest coefficient of 0.81 (CI = 0.79–0.83), indicating a high level of temporal stability over this period.

In the following analysis, we evaluated the convergent and divergent validity of the SRQ's subscales.

Correlations of academic burnout and academic support scores assessed the convergent validity of the SRQ. Positive correlations of subscales of the SRQ with academic burnout and academic support ranging from 0.28 to 0.59 indicated acceptable convergent and divergent validity (Table 5).

**Table 5:** Pearson correlation between SRQ’ subscales with academic burnout and academic support

	Academic Burnout	Academic Support
Teacher's Skills Creating a Resilient Classroom	-0.53^**^	0.55^**^
Collaborative Environment	-0.44^**^	0.48^**^
Departmental Security Environment	-0.39^**^	0.43^**^
Intimate Environment	-0.41^**^	0.37^**^
Respectful Environment	-0.28^**^	0.33^**^
Legal Environment	-0.55^**^	0.59^**^

< 0.05 **p < 0.01

## Discussion

The primary objective of this research was to develop and conduct a psychometric assessment of a novel questionnaire, namely the School Resilience Questionnaire (SRQ), designed to measure the level of resilience within Iranian adolescents' educational institutions. Given the growing recognition of the importance of resilience in promoting well-being and academic success, it is crucial to have a valid and reliable measure specifically tailored to the Iranian context. Through an Expert Panel process, a comprehensive scale comprising 55 items was developed, considering the unique cultural and educational factors in Iran. This process ensured the SRQ was culturally appropriate and sensitive to the experiences of Iranian adolescents. Our sample consisted of 510 adolescent students. For the EFA phase, 200 adolescent students were selected from various educational schools. In the CFA phase, 310 adolescent students were used. The factor structure of the scale was examined through both EFA and CFA. The EFA analysis revealed six consistent and robust factors across the entire sample. These factors were identified as Teacher's Skills in Creating a Resilient Classroom, Collaborative Environment, Departmental Security Environment, Intimate Environment, Respectful Environment, and Legal Environment. Each factor represents a unique aspect of the educational environment that contributes to the development of school resilience among Iranian adolescents. Based on the CFA results, all the items in the scale exhibited significant and acceptable factor loadings. Additionally, the CFA analysis indicated that the six-factor structure of the scale demonstrated a good fit to the collected data. To establish the construct validity of the scale, fit statistics were computed and compared against predefined criteria. The results indicated strong construct validity for the SRQ, suggesting that it effectively measures resilience within Iranian educational institutions. Additionally, the scale demonstrated satisfactory internal consistency, as evidenced by high reliability coefficients such as Cronbach's alpha.

For establishing the convergent validity of the School Resilience Questionnaire (SRQ)'s subscales, we assessed their relationship with measures of academic support. Convergent validity assesses the positive correlation between related constructs. Thus, our objective was to investigate whether the subscales of the School Resilience Questionnaire (SRQ) would demonstrate a positive association with indicators of academic support. These findings align with the existing literature and studies have shown that academic support have a significant positive impact on students' resilience and academic outcomes [31–33]. Academic support plays a crucial role in promoting School Resilience by providing students with tailored assistance, resources, and guidance. This could effectively address students' academic needs and challenges, equipping them with the necessary skills, knowledge, and strategies to overcome obstacles and enhance their resilience [34]. Moreover, academic support offers valuable opportunities for students to cultivate positive relationships with peers, mentors, and educators, further bolstering their sense of belonging and resilience within the school environment. A resilient school environment enhances the academic support, as students thrive in an atmosphere that promotes their well-being and growth [35].

In order to evaluate the divergent validity of the SRQ's subscales, we analyzed their relationship with academic burnout. Our aim was to ascertain that the SRQ's subscales would exhibit a significant correlation with indicators of academic burnout, indicating a distinct and divergent relationship between these constructs. As results, the negative relationship suggests that as levels of School Resilience increase, levels of academic burnout tend to decrease, and vice versa. In other words, higher levels of School Resilience are associated with lower levels of academic burnout.

This finding aligns with the existing literature [36, 37], which consistently demonstrates that School Resilience serves as a protective factor against academic burnout. Schools that possess higher levels of resilience are better equipped to provide support to students in coping with academic challenges, setbacks, and stressors. By cultivating an environment that fosters adaptability, motivation, and perseverance, these schools contribute to reducing the likelihood of students experiencing academic burnout. In contrast, schools with lower levels of resilience may have students who are more vulnerable to academic burnout [38]. These students often struggle to effectively manage the demands of academics, lack motivation and confidence, and feel overwhelmed by stressors. Consequently, these factors significantly contribute to elevated levels of academic burnout, which detrimentally influences students' overall academic well-being and performance.

By conducting these analyses, we aimed to provide evidence of the convergent and divergent validity of the SRQ's subscales. This would affirm the questionnaire's ability to effectively measure resilience while differentiating it from related constructs, such as academic support and academic burnout. It is important to note that the measures of academic support and academic burnout were obtained using validated scales specifically designed for assessing these constructs.

The present findings hold significant implications for educational institutions and resilience interventions in Iran. By using the SRQ, educators, and policymakers can gain valuable insights into the specific areas where interventions and support systems can be implemented to enhance students' and schools' resilience and promote their well-being and academic achievement.

This study will also be considered in the context of the following limitations: One limitation of the study is the recruitment of participants exclusively from 15 specific local areas in Tehran, which might potentially limit the generalizability of the findings to schools across Iran. To mitigate this limitation, future research endeavors could strive to obtain a more representative sample of schools from various regions in the country. This could be achieved through collaboration with multiple educational institutions nationwide, ensuring a broader representation of schools in the study. By incorporating schools from different regions, the findings can be more effectively extrapolated to the larger population of schools in Iran. Another limitation pertains to the temporal scope of the study, which was confined to the academic year 2021–2022. The generalizability of the findings to other time periods may be constrained. To address this limitation, future research could adopt a longitudinal design encompassing multiple academic years, thus capturing potential variations in school resilience over time. Such an approach would yield a more dynamic understanding of how school resilience evolves and fluctuates among Iranian adolescents. Furthermore, the study relied exclusively on self-assessment scales as measurement instruments. To surmount this limitation, future studies could consider incorporating multiple measurement approaches. This might involve objective observations, interviews with teachers, parents, or school staff, or the utilization of standardized assessments. By employing a combination of diverse measurement methods, researchers can obtain a more comprehensive and well-rounded assessment of school resilience, reducing potential measurement bias and providing a more accurate depiction of the construct. In addition, the study may have primarily focused on a specific age range or grade level of Iranian adolescents. To enhance the generalizability of the findings, future research should aim to encompass a broader range of ages and educational levels, spanning from early adolescence to late adolescence. This would yield a more comprehensive understanding of how school resilience develops and manifests across different stages of adolescence, facilitating more nuanced conclusions and recommendations.

In light of the study's limitations, several recommendations can be made for future research endeavors. Firstly, efforts should be directed towards obtaining a more representative sample of schools from diverse regions across Iran, thus enhancing the generalizability of findings. Longitudinal studies covering multiple academic years should be considered to capture the dynamic nature of school resilience among Iranian adolescents. Diversifying measurement methods beyond self-assessment scales, such as incorporating objective observations and standardized assessments, can provide a more comprehensive evaluation of school resilience. Moreover, future research should aim to include a broader age range of adolescents, from early to late adolescence, to gain a deeper understanding of how school resilience evolves across different developmental stages. Lastly, researchers are encouraged to explore the clinical applications of any developed questionnaires or tools, including their potential use in early identification, targeted interventions, and monitoring of students at risk.

In engaging with the existing literature, this study's findings align closely with previous research that underscores the significance of school resilience in adolescent development and academic outcomes. The robust factors identified within the SRQ, such as 'Teacher's Skills in Creating a Resilient Classroom' and 'Collaborative Environment', resonate with the findings of similar studies which highlight the critical role of teachers and peer support in fostering resilience [39, 40]. Our research extends these concepts by offering a culturally nuanced perspective, particularly relevant to the Iranian educational context. The strong correlation between school resilience and academic success, as evidenced in our study, is consistent with the previous research [41]. It also showed a positive link between these two variables in their research. Furthermore, our findings about the inverse relationship between school resilience and academic burnout contribute to the growing body of evidence that supports the protective role of resilience against academic stressors [42]. By providing empirical support to these established theories, our study not only reaffirms the importance of resilience in educational settings but also enhances our understanding of its specific dynamics within Iranian schools. This contributes to a more comprehensive framework for developing targeted interventions and policies aimed at boosting resilience and academic success among adolescents in Iran.

In conclusion, the present study has successfully developed and validated the 55-item School Resilience Questionnaire (SRQ) for implementation among Iranian adolescents. The findings robustly demonstrate that the SRQ exhibits a high level of internal consistency, reliability, temporal stability, and strong validity, establishing it as a reliable and valid measurement tool. The inclusion of diverse factors within the questionnaire enables a comprehensive assessment of school resilience, providing valuable insights for both educational research and practice. The development and validation of the SRQ contribute significantly to the field of school resilience assessment, underscoring its importance. It is our sincere expectation that the SRQ will serve as an invaluable instrument for researchers and practitioners alike, facilitating a deeper comprehension of school resilience and enabling the implementation of evidence-based practices to support the well-being and academic achievement of adolescents in the educational setting.

### Supplementary Information

**Additional file 1.**

## Data Availability

The corresponding author will provide the datasets generated and analyzed during this study upon a reasonable request.
