# Supplementary material for: Development and validation of School Resilience Questionnaire (SRQ) in Iranian adolescents
Source: BMC Psychol. 2024 Jan 18;12:31. doi: 10.1186/s40359-023-01496-6 (PMC10795288; doi:10.1186/s40359-023-01496-6)
Supplement: Supplementary file 1 — Additional file 1. [file 40359_2023_1496_MOESM1_ESM.docx]

1= It is not true at all. 5= It is perfectly true.

| 5 | 4 | 3 | 2 | 1 | Questions | No |
| --- | --- | --- | --- | --- | --- | --- |
|  |  |  |  |  | Students feel free at school. | 1 |
|  |  |  |  |  | Students actively participate in class activities. | 2 |
|  |  |  |  |  | Teachers assist students in successfully completing activities. | 3 |
|  |  |  |  |  | Teachers provide additional support to students who need it. | 4 |
|  |  |  |  |  | Teachers are supportive and encouraging, fostering a positive learning environment. | 5 |
|  |  |  |  |  | Teachers pay attention to and nurture each student's abilities. | 6 |
|  |  |  |  |  | Trust in students' abilities is a hallmark of our teaching approach. | 7 |
|  |  |  |  |  | Due to the teachers' encouragement, I feel motivated to exert greater effort. | 8 |
|  |  |  |  |  | A close and respectful relationship exists between teachers and students. | 9 |
|  |  |  |  |  | Students feel safe and calm in the classroom. | 10 |
|  |  |  |  |  | Teachers set achievable and reasonable expectations, tailored to students' abilities. | 11 |
|  |  |  |  |  | The school's ethos is grounded in fairness and equality. | 12 |
|  |  |  |  |  | In our school, humiliation and negative labeling of students are absent. | 13 |
|  |  |  |  |  | Students consistently receive support and security from adults at the school. | 14 |
|  |  |  |  |  | I feel comfortable and secure within the school environment. | 15 |
|  |  |  |  |  | The school maintains fixed, clear rules and boundaries. | 16 |
|  |  |  |  |  | Our school's rules are explicit and unambiguous. | 17 |
|  |  |  |  |  | Students have many opportunities for success and personal growth. | 18 |
|  |  |  |  |  | I experience a strong sense of care and support at school. | 19 |
|  |  |  |  |  | Specialized services are available for disadvantaged students, addressing their needs respectfully. | 20 |
|  |  |  |  |  | The school offers a variety of tours, sports programs, and cultural festivals. | 21 |
|  |  |  |  |  | Extracurricular recreational programs enhance student life. | 22 |
|  |  |  |  |  | Positive relationships among students are evident, with a spirit of support and cooperation. | 23 |
|  |  |  |  |  | Classrooms often use collaborative groups to enhance learning. | 24 |
|  |  |  |  |  | Students look out for each other in all activities. | 25 |
|  |  |  |  |  | Teachers effectively resolve classroom issues and manage tensions. | 26 |
|  |  |  |  |  | Teachers are adept at making appropriate decisions in challenging situations and maintaining classroom order. | 27 |
|  |  |  |  |  | The school environment is free from coercion and threats. | 28 |
|  |  |  |  |  | School rules are developed through a collaborative process. | 29 |
|  |  |  |  |  | The school environment allows for enjoyment and fun. | 30 |
|  |  |  |  |  | Discriminatory behavior has no place in our school. | 31 |
|  |  |  |  |  | Staff interactions with students are consistently empathetic. | 32 |
|  |  |  |  |  | Teachers adopt empathetic approaches in their interactions with students. | 33 |
|  |  |  |  |  | The implementation of rules is free from destructive methods and humiliating punishments. | 34 |
|  |  |  |  |  | The school fosters a low-competition environment, avoiding unfavorable comparisons among students. | 35 |
|  |  |  |  |  | Academic counseling is regularly offered, tailored to each student's interests and abilities. | 36 |
|  |  |  |  |  | The school places high importance on problem-solving and the development of thinking skills. | 37 |
|  |  |  |  |  | Students receive guidance in managing their emotions. | 38 |
|  |  |  |  |  | Life skills are a key focus of our educational approach. | 39 |
|  |  |  |  |  | There is no coercion or threats in the school. | 40 |
|  |  |  |  |  | School rules are developed collaboratively. | 41 |
|  |  |  |  |  | It is possible to have fun at school. | 42 |
|  |  |  |  |  | There is no discriminatory behavior in the school. | 43 |
|  |  |  |  |  | The behavior of staff with students is empathetic. | 44 |
|  |  |  |  |  | The teacher's behavior with the students is empathetic. | 45 |
|  |  |  |  |  | No destructive methods are used to implement the rules. | 46 |
|  |  |  |  |  | There are no humiliating punishments in the school. | 47 |
|  |  |  |  |  | Competition at school is low. | 48 |
|  |  |  |  |  | Students are not compared to the same person. | 49 |
|  |  |  |  |  | Academic counseling is offered to students regularly. | 50 |
|  |  |  |  |  | Academic guidance is in line with the student's interests and abilities. | 51 |
|  |  |  |  |  | Problem-solving is emphasized in school. | 52 |
|  |  |  |  |  | The school focuses on improving students' thinking skills. | 53 |
|  |  |  |  |  | Students are supported in managing their emotions. | 54 |
|  |  |  |  |  | Emphasis is placed on teaching life skills in the school. | 55 |
